# Rapid Assessment Tool for *Haemophilus influenzae* type b Disease in Developing Countries^1^

**DOI:** 10.3201/eid1007.030737

**Published:** 2004-07

**Authors:** Daniel R. Feikin, Christopher B. Nelson, James P. Watt, Ezzeddine Mohsni, Jay D. Wenger, Orin S. Levine

**Affiliations:** * Centers for Disease Control and Prevention, Atlanta, Georgia, USA;; † World Health Organization, Geneva, Switzerland;; ‡ Johns Hopkins Bloomberg School of Public Health, Baltimore, Maryland, USA;; § World Health Organization, Cairo, Egypt

**Keywords:** Haemophilus influenzae type b, meningitis, pneumonia, conjugate vaccines, developing countries, research

## Abstract

*Haemophilus influenzae* type b (Hib) still causes a substantial number of deaths among children in developing countries, despite the availability of effective conjugate vaccines. A major obstacle in developing a Hib vaccine has been limited awareness about the impact of Hib disease. A tool was developed to estimate the national rates of Hib meningitis and pneumonia by assessing retrospective local data over 7 to 10 days. Data from 11 countries in Africa, the Middle East, and Asia were studied and showed rates of Hib meningitis from >50 cases per 100,000 children >5 years in Ghana and Uganda to <15 per 100,000 in Iran, Jordan, and Uzbekistan. Results were affected by the quality of available data. The Hib rapid assessment tool can be useful to countries that desire a timely assessment of Hib disease rates.

## Abstract 2

*Haemophilus influenzae* type b disease prevalence in children provides estimates of national disease prevalence.

Among infants and young children, *Haemophilus influenzae* type b (Hib) is the leading cause of bacterial meningitis deaths and the second leading cause of bacterial pneumonia deaths worldwide and accounts for approximately 400,000 deaths of children each year (*1**,**2*). These deaths are preventable through Hib conjugate vaccines (*1*). In the United States, Hib conjugate vaccines were first introduced into the routine immunization program for infants in 1990. Subsequently, most industrialized, western countries have introduced these vaccines. Hib conjugate vaccines, however, are still not widely used in developing countries, where the greatest rates of Hib-related disease and deaths occur. Before 2002, children in only 2 of 51 countries with an infant death rate >70 per 1,000 live births were routinely vaccinated against Hib (Figure 1).

**Figure 1:**
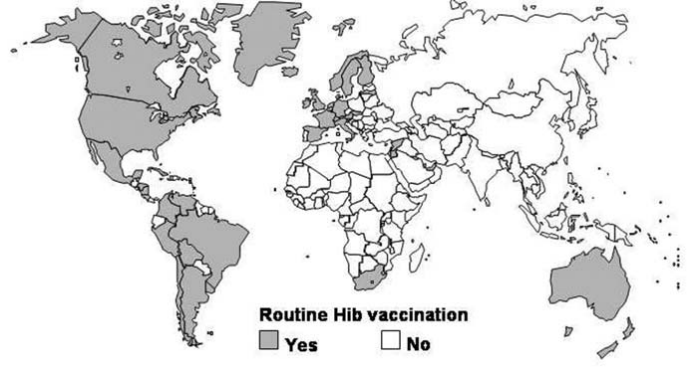
Global status of countries using Hib conjugate vaccine in their national immunization program in 2001 (J. Wenger, WHO, unpub. data).

The major obstacles to introducing Hib conjugate vaccines into developing countries have been their cost and the population’s limited awareness about the impact of Hib disease. The Hib conjugate vaccine, even after a decade of use, is still expensive, costing approximately $U.S. 2.50 per dose, and thus, external financial resources are needed to procure the vaccine in most developing countries. Since 1999, the Global Alliance for Vaccines and Immunization (GAVI) through the Vaccine Fund has provided a mechanism of financing Hib conjugate vaccines for 5 years to the 75 poorest countries (http://www.vaccinealliance.org/home/index.php). Despite this opportunity, few countries initially requested the Hib vaccine through GAVI, partly because of limited awareness about the rate of Hib disease.

Diagnosing Hib disease is difficult; Hib primarily causes meningitis and pneumonia, two common syndromes often treated empirically. To diagnose Hib meningitis, lumbar punctures must be performed and cerebrospinal fluid rapidly processed in a microbiologic laboratory with the technical capability and supplies to culture Hib. Hib pneumonia is even more difficult to diagnose. Although blood cultures are highly specific for Hib, they have a sensitivity of 20%, thus allowing the role of Hib as a cause of pneumonia to be underestimated (*3**,**4*).

Several methods have been used to define the rate of Hib disease in developing countries. Randomized, controlled trials of the Hib conjugate vaccine in The Gambia, which looked at radiographic evidence of pneumonia as a study endpoint, showed that approximately 20% of consolidated pneumonia (diagnosed by chest x-ray) is prevented by vaccination and therefore presumably caused by Hib (*5*). In Chile, a retrospective analysis of pneumonia after a randomized, controlled trial of Hib vaccine showed a 22% reduction in consolidation or pleural effusion in the group that received vaccine (*6*). Despite the quality of the data produced, such trials are too expensive, time-consuming, and complex to be widely used to measure Hib disease. A more widely used method to measure Hib disease has been population-based laboratory surveillance for Hib meningitis (*7*). Such surveillance, although dependent on the rate of lumbar punctures and laboratory quality, can produce reliable data on the incidence rate of Hib meningitis, which can be compared across countries. Population-based surveillance for meningitis, however, requires at least a year to produce results and does not give an estimate of Hib pneumonia.

Alternatives to these longitudinal, time-consuming, resource-intense methods of measuring Hib disease are being sought, particularly for use in regions of the world, such as Africa, where studies have consistently shown high rates of Hib disease (*8**–**12*). In countries in these regions, more limited data on the local rate of Hib disease may be adequate to allow evidence-based decisions about introducing Hib vaccine and to provide a basis for national advocacy for, and commitment to, such decisions.

On the basis of these considerations, we created a rapid assessment tool to measure the national rate of severe Hib disease. We discuss the methods used in the rapid assessment tool and its results in the first 11 countries where it was used.

## Methods

### Logistics

Requests for Hib rapid assessments were made by national ministries of health. The Hib rapid assessment tool was usually completed in 7 to 10 days. The assessment teams consisted of local representatives from the ministry of health and one or two consultants familiar with the tool. National data relevant to the tool was collected by reviewing national health statistics and medical literature, focusing on national or regional journals. The assessment often included several days in the capital city reviewing available data and working with the ministry of health. Most of the assessment involved collecting, evaluating, and synthesizing existing data from hospitals. Data were entered into standardized spreadsheets that assist in the calculation of the national disease impact. The assessment often ended with a meeting to discuss the findings with key persons in the ministry of health and leading pediatricians.

The tool uses two different, complementary methods to estimate Hib rates. The first one works from the "bottom up," beginning with a local estimate of Hib meningitis incidence rates; the second works from the "top down," starting with the country’s under-5 mortality rate, which is the number of children per 1,000 live births who die before their fifth birthday.

### Meningitis Incidence Rate Method

This method uses retrospective data to calculate an incidence rate of Hib meningitis, from which the annual numbers of cases of and deaths attributable to Hib meningitis and pneumonia are estimated. The first step for this method is selecting an appropriate site where the incidence of Hib meningitis can be accurately calculated. The ideal site is a region of the country where the catchment population of a hospital or a few hospitals can be well defined, where most children with meningitis go to these hospitals, and where the private sector rarely uses the Hib vaccine. In addition, physicians in these hospitals should routinely perform lumbar punctures on children with suspected meningitis, and the hospitals should have laboratories that culture *H. influenzae* and conduct cytologic tests on cerebrospinal fluid. Although few laboratories in our assessments performed serotyping, we assumed that, in the absence of vaccination, all *H. influenzae* in cerebrospinal fluid was caused by type b (Hib), which is consistent with available studies (*13**,**14*).

**Figure 2:**
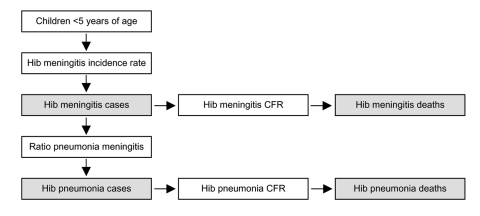
The meningitis incidence rate method for calculating *Haemophilus influenzae* type b (Hib) disease rate by using the Hib rapid assessment tool. White boxes are data input points and gray boxes are disease rate estimates.

Calculating Hib meningitis incidence rate begins by counting the number of cases of culture-confirmed meningitis in children <5 years of age during a defined period in a defined catchment population (Figure 2). The percentage of culture-confirmed meningitis caused by Hib is then calculated. Cerebrospinal fluid samples from neonates are excluded because Hib is very rare in the first month of life (*15*). Next, the number of purulent cerebrospinal fluid samples during the same period is counted. For the purposes of this tool, purulence is defined in a way that increases the specificity for bacterial meningitis: visible turbidity, >100 leukocytes/mm^3^, or 10–99 leukocytes/mm^3^ with glucose <40 mg/dL and protein >100 mg/dL. The culture-confirmed cases are excluded, which leaves a count of culture-negative, purulent cerebrospinal fluid samples. The tool assumes that these are bacterial in origin, and the number ascribed to Hib is calculated by applying the same percentage of culture-confirmed meningitis caused by Hib to the culture-negative, purulent specimens. The rationale for this step is that a certain percentage of Hib meningitis cases may not be culture-confirmed because of prior antimicrobial drug use or laboratory-related factors. The number of culture-confirmed Hib cases and estimated culture-negative Hib cases is added. Then this number is inflated by a factor on the basis of information from local pediatricians to account for children with suspected meningitis who did not get a lumbar puncture. The number of children <5 years of age in the catchment region of the hospitals is used as the denominator to calculate the Hib meningitis incidence rate.

In the last step, the total number of children <5 years of age in the country is used to estimate the annual number of cases of Hib meningitis in the country. By using a ratio of five Hib pneumonia cases for each case of Hib meningitis, which was found in two randomized studies of Hib conjugate vaccine, the annual number of cases of Hib pneumonia is estimated (*5**,**6*). By using the case-fatality proportions of Hib meningitis and Hib pneumonia, obtained locally if possible, the annual number of deaths attributable Hib in the country is also estimated.

### Under-5 Mortality Rate Method

**Figure 3:**
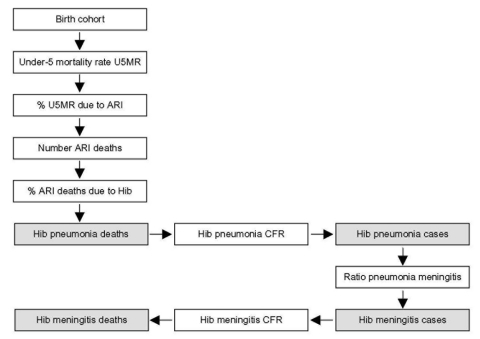
The under-5 mortality rate method for calculating *Haemophilus influenzae* type b (Hib) disease rate by using the Hib rapid assessment tool. White boxes are data input points and gray boxes are disease rate estimates.

This method starts with the birth cohort in the country (Figure 3). To calculate the number of childhood deaths in the country, the under-5 mortality rate is applied to the birth cohort. The neonatal death rate, if available, is subtracted from this number. Then the percentage of childhood deaths caused by acute respiratory illness is estimated. This percentage is extrapolated from a study that showed that the higher the under-5 mortality rate, the greater percentage of deaths that are caused by respiratory illness (*16*). In the next step, the percentage of all acute respiratory illness deaths due to Hib is estimated to be 13%. This figure was obtained by subtracting the number of respiratory deaths unlikely to be caused by Hib, during the neonatal period and caused by measles and pertussis, and multiplying the remaining number of respiratory deaths by 20%, the percentage of severe pneumonia thought to be caused by Hib (*5**,**6**,**17*). This calculation yields the annual number of pneumonia deaths caused by Hib. Then, meningitis cases and meningitis deaths are calculated by using the case-fatality proportion of Hib pneumonia, the 5:1 Hib pneumonia:meningitis ratio, the Hib meningitis case-fatality proportion, and the annual number of Hib pneumonia cases.

### Review of the Rapid Assessment Tool

The tool was pilot-tested in several countries, and a draft version was reviewed at a meeting of technical experts convened by the World Health Organization in October 2000. Suggested revisions to the tool made at the meeting were subsequently incorporated. The minutes of this meeting (http://www.who.int/vaccines-documents/DocsPDF01/www604.pdf), along with the completed tool including worksheets, are available on the Internet (http://www.who.int/vaccines-documents/DocsPDF01/www625.pdf).

## Results

The Hib rapid assessment tool has been used in 11 developing countries in sub-Saharan Africa, the Middle East, and Asia (Table 1 and 2). The assessment of the meningitis incidence rate was performed most often in smaller cities, where the denominator population could be more easily delineated, although several assessments were performed in larger cities, such as Alexandria, Egypt, and Shiraz, Iran. In most countries, at least one hospital had cultured *H. influenzae* from cerebrospinal fluid. The exceptions were Uzbekistan, Yemen, and Ghana. Alternative strategies were used to estimate the incidence rate of Hib meningitis in these countries. In Uzbekistan, where laboratories had been unable to culture Hib from cerebrospinal fluid and the leukocyte counts were not being performed properly, data from a prospective, population-based study in Moscow, which has a very similar health care system, were applied to the rate of lumbar punctures performed in Uzbek children to yield an estimate of the Hib meningitis incidence (A. Platonov, pers. comm.). In Ghana, Gram stain results of cerebrospinal fluid, rather than culture, were used to estimate the rate of Hib meningitis. In Yemen, data about purulent meningitis from another hospital in the capital and review of data from the local literature were used to estimate the rate of Hib meningitis.

**Table 1:** Results of *Haemophilus influenzae* type b (Hib) rapid assessment tool (meningitis incidence rate method) in 11 developing countries

Country	% culture-confirmed^a^	Unadjusted Hib meningitis rate^b^	Adjusted Hib meningitis rate^c^	Heningitis cases/deaths^d^	Pneumonia cases/deaths^d^
Ghana	NA	0	72^e^	2,030/609	10,148/2,030
Uganda	50	44	59	2,533/633	12,663/759
Egypt	50	1	23	1,795/682	8,977/539
Iran	67	5	7	599/12	2,994/180
Jordan	43	6	14	107/5	534/27
Morocco	44	6	23	689/34	3,443/172
Oman	43^f^	27	41	112/11	560/34
Yemen	NA	0	23^f^	790/245	3,950/897
Kyrgyzstan	33	15	20	101/12	505/51
Uzbekistan	NA	0	4^g^	103/12	515/52
Bhutan	42	6	15	16/5	80/8

^a^Meningitis caused by Hib. ^b^Unadjusted meningitis incidence rate is the number of culture-confirmed Hib meningitis cases per 100,000 children <5 years of age in the study site without making any of the adjustments accounted for in the rapid assessment tool (see Methods). ^c^Adjusted meningitis incidence rate is the number of Hib meningitis cases per 100,000 children <5 years of age in the study site, as determined by the rapid assessment tool (see Methods). ^d^National estimates. ^e^Based on Gram stain of coccobacilli. ^f^Based on literature review and use of data from hospital. ^g^Based on use of data from Moscow study and rate of lumbar puncture in Uzbekistan.

**Table 2:** Results of *Haemophilus influenzae* type b (Hib) rapid assessment tool (under-5 mortality rate method) in 11 developing countries

Country	U5MR^a^	Meningitis cases/deaths^a^	Pneumonia cases/deaths^b^	Ratio meningitis cases U5MR:MIR^a^
Ghana	110	5,465/984	27,326/4,099	2.7
Uganda	147	2,838/709	14,189/2,838	1.1
Egypt	65	6,731/2558	33,657/2,019	3.7
Iran	31.5	1,975/40	9,876/593	3.3
Jordan	33	313/16	1,563/78	2.9
Morocco	Not done			–
Oman	21.5	48/15	242/14	0.43
Yemen	105	3,085/956	15,427/956	3.9
Kyrgyzstan	19.6^c^	125/15	625/63	1.2
Uzbekistan	21^c^	565/68	2,826/283	5.4
Bhutan	63^c^	54/18	270/27	3.4

^a^U5MR, under-5 mortality rate method; MIR, meningitis incidence rate method. ^b^National estimates. ^c^U5MR excludes neonatal disease.

The percentage of culture-confirmed meningitis caused by Hib was 33%–67%. Most hospitals were able to perform leukocyte counts on cerebrospinal fluid, but a few hospitals routinely measured protein and glucose. The incidence rate of culture-confirmed Hib meningitis varied greatly, depending on the ability to find hospitals consistently able to culture Hib from cerebrospinal fluid. Except for a few countries (i.e., Uganda, Oman, and Kyrgyzstan), the rates of culture-confirmed meningitis were very low, <10 per 100,000 children <5 years of age. When we used the rapid assessment tool, the estimated rates of Hib meningitis tended to be several-fold higher than those based on culture-confirmed Hib. When we used the tool, the estimated incidence rate of Hib meningitis among children <5 years of age was >50 cases per 100,000 children <5 years of age in Ghana and Uganda to <15 cases in Iran, Jordan, and Uzbekistan. The under-5 mortality rate method yielded estimates of national Hib disease rates that were higher than those obtained from the meningitis incidence rate method (mean 2.8, 95% confidence interval 1.7–3.9.)

## Discussion

**Figure 4:**
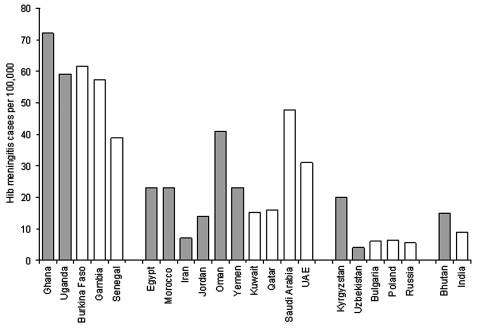
Comparison of incidence rates of *Haemophilus influenzae* type b (Hib) meningitis per 100,000 children <5 years of age between the rapid assessment tool (gray bars) and prospective, population-based laboratory surveillance (white bars), by region (*8**–**10**,**12**,**19**–**26*). (UAE, United Arab Emirates).

The Hib rapid assessment tool estimated rates of Hib meningitis in Africa and the Middle East that were similar to those obtained from population-based studies in the same regions (Figure 4 8–12,18–22, ). In countries in Eastern Europe and Asia where the impact of Hib meningitis is less well-defined, the tool yielded variable results, although not too discrepant from rates found from prospective, population-based surveillance performed in other countries in the same areas (*23**–**26*). These results demonstrate that the Hib rapid assessment tool can yield estimates of Hib disease rates similar to expected rates in many countries. Without some of the adjustments made by the tool, the incidence rate of culture-confirmed Hib meningitis would have been low in most of these countries. One strength of the tool is that its adjustments compensate for artificial reasons for low incidence rates of culture-confirmed Hib meningitis, such as low rates of lumbar puncture and the lack of appropriate microbiologic capacity.

While not meant to replace more rigorous and accurate methods used to measure Hib disease, such as prospective, population-based surveillance, the tool addresses a unique need for countries desiring local Hib disease rate estimates to assist them in making a decision to introduce Hib vaccine. The strengths of the tool in addressing this need are that it is rapid, inexpensive, uses locally obtained data, accounts for biases in these data that might underestimate the true rate of Hib disease, and provides disease impact output as the number of cases and deaths from Hib disease, which is often more tangible to decision makers than incidence rates. Moreover, the process of performing the rapid assessment engages pediatricians and decision makers in a dialogue about the clinical, epidemiologic, and laboratory aspects of Hib disease and vaccine. Consequently, the Hib rapid assessment process helps countries develop the capacity to critically evaluate existing local data and perceptions about Hib disease rates.

The Hib rapid assessment tool has several limitations. Whereas in most countries we found hospitals that effectively cultured Hib, in several countries we were unable to find any site with demonstrated capacity to culture Hib. In these countries, we were unable to use the meningitis incidence rate method as outlined in the tool. As mentioned, alternative strategies based on local and regional data were used here, which might have introduced inaccuracies. Nonetheless, the estimates of Hib impact in these countries were similar to those observed in population-based studies from the same regions (Figure 4).

A second limitation of the tool is that even when a hospital that has cultured Hib is found, the output from the tool using the meningitis incidence rate method will always be constrained by the quality of the data. Although the tool corrects for missed cases of Hib meningitis, it does not correct completely for substantial underdiagnosis of Hib meningitis. If clinical or microbiologic factors have led to such underdiagnosis, this will necessarily be reflected in the rate estimates made by the tool. This limitation was exemplified during the rapid assessment in Kyrgyzstan, where the tool found a rate of Hib meningitis of 20.4 per 100,000 in 1999 to 2000, a period when the infectious disease hospital in Bishkek was involved in a study with Aventis-Pasteur, which supplied the laboratory with appropriate reagents and materials to isolate Hib. From 2001 through 2002, after the study ended, the rate obtained from tool in the same hospital decreased to 4.7 per 100,000.

A third limitation of the rapid assessment tool is that several assumptions are incorporated into the calculations. These assumptions, although based on the available literature, may not apply to all populations. For example, the 5:1 ratio of Hib pneumonia to meningitis, which was based on the results of two randomized controlled vaccine trials in The Gambia and Chile, may not apply to all parts of the world (*5**,**6*). In countries with lower under-5 mortality rates, the 5:1 ratio may be too high, while in countries with high under-5 mortality rates, the 5:1 ratio may be too low. Moreover, this ratio may vary in different parts of the world, as suggested from the results of a recently completed Hib conjugate vaccine efficacy trial in Lombok, Indonesia, which suggested the incidence of Hib meningitis to be high and the incidence of radiographically defined Hib pneumonia to be relatively low (*27*).

A fourth concern is that the under-5 mortality rate method yielded higher estimates of Hib disease rates than the meningitis incidence rate method. One reason for this difference is that the neonatal death rate was not subtracted from the under-5 mortality rate in most countries (except Kyrgyzstan, Uzbekistan, and Bhutan). This omission may have led to overestimating Hib disease since neonatal deaths account for a large proportion of childhood deaths, but Hib deaths are rare in neonates (*13*). Also, because the under-5 mortality rate method starts with pneumonia deaths and works backwards to meningitis cases, more assumptions are made in the last estimate. Therefore, in reconciling the estimates obtained from the two methods, the meningitis prevalence may be more accurate than the meningitis incidence rate method, while estimation of the pneumonia incidence may have an accuracy in between those obtained from the two methods.

The results of the Hib rapid assessment tool need to be interpreted in light of its limitations and the validity of its assumptions critically evaluated in each country where it is used, particularly in regions, such as Asia, where the impact of Hib disease is still uncertain. Nonetheless, the tool has been useful to some countries in deciding whether to adopt Hib vaccine into their national immunization programs. Of the countries we studied, three (Ghana, Uganda, and Oman) introduced Hib vaccine, and three others (Bhutan, Kyrgyzstan, and Yemen) requested Hib vaccine on their initial application to GAVI. Subsequent to the assessments in these countries, the tool has been used in several other developing countries that were considering the introduction of Hib vaccine (Western Pacific Islands [28], Albania, Thailand, Nepal, and Sudan). Several countries (Uganda, Albania, and Egypt) have used results of the rapid assessment tool in cost-effectiveness analyses of Hib vaccine introduction. As more developing countries consider Hib vaccination programs, estimating the local impact of Hib disease will continue to be an important part of the effort to introduce and sustain Hib vaccination programs. The experience with measuring Hib disease rates, including the rapid assessment tool, might also provide a valuable base for considering the introduction of other new vaccines, including pneumococcal and meningococcal conjugates.
